# Predictive genotype-phenotype relations using genetic diversity in African yam bean (*Sphenostylis stenocarpa* (Hochst. ex. A. Rich) Harms)

**DOI:** 10.1186/s12870-021-03302-0

**Published:** 2021-11-20

**Authors:** Ademola Aina, Ana Luísa Garcia-Oliveira, Christopher Ilori, Peter L. Chang, Muyideen Yusuf, Olaniyi Oyatomi, Michael Abberton, Daniel Potter

**Affiliations:** 1 University of Ibadan, Ibadan, Nigeria; 2 International Institute of Tropical Agriculture, Ibadan, Nigeria; 3 Excellence in Breeding (EiB), CIMMYT-ICRAF, UN Av., Nairobi, Kenya; 4 Dept. Mol. Biology, Biotechnology & Bioinformatics, CCS Haryana Agricultural University, Hisar, Haryana India; 5 University of California, Davis, USA; 6 University of Southern California, Los Angeles, USA

**Keywords:** Africa yam bean, Genetic diversity, RAD SNP markers, Genotyping, Phenotype

## Abstract

**Background:**

African Yam Bean (AYB) is an understudied and underutilized tuberous legume of tropical West and Central African origin. In these geographical regions, both seeds and tubers of AYB are important components of people’s diets and a potential target as a nutritional security crop. The understanding of the genetic diversity among AYB accessions is thus an important component for both conservation and potential breeding programs.

**Results:**

In this study, 93 AYB accessions were obtained from the International Institute of Tropical Agriculture (IITA) genebank and genotyped using 3722 SNP markers based on Restriction site-Associated DNA sequencing (RAD-Seq). Genetic data was analysed using multiple clustering methods for better understanding the distribution of genetic diversity across the population. Substantial genetic variability was observed in the present set of AYB accessions and different methodologies demonstrated that these accessions are divided into three to four main groups. The accessions were also analysed for important agronomic traits and successfully associated with their genetic clusters where great majority of accessions shared a similar phenotype.

**Conclusions:**

To our knowledge, this is the first study on predicting genotypic-phenotypic diversity relationship analysis in AYB. From a breeding perspective, we were able to identify specific diverse groups with precise phenotype such as seed or both seed and tuber yield purpose accessions. These results provide novel and important insights to support the utilization of this germplasm in AYB breeding programs.

**Supplementary Information:**

The online version contains supplementary material available at 10.1186/s12870-021-03302-0.

## Background

African yam bean (*Sphenostylis stenocarpa* Hochst. ex. A. Rich. Harms, Fabaceae) is an understudied and underutilized tuberous legume of tropical Africa [1]. Among the seven existing species in the genus *Sphenostylis,* African yam bean (AYB) is most economically important species [2]. AYB is well appreciated by few farmers who in time past had depended on this indigenous crop as a cheap source of protein, where both tubers and pods are edible. African yam bean, though a well-adapted crop with excellent nutritional potential; still is classified as a neglected and underutilized species [3]. Limitations to the cultivation of AYB include undesirable plant physiological as well organoleptic characteristics. At the physiological level, this crop is characterized by having photoperiodic sensitivity and longer maturity period [4–6], that lead to poor productivity. Moreover, its hard seed coat prolongs cooking time that is translated into time-money losses [4, 5]. Additionally, the low availability of seeds, lack of staking material, post-harvest diseases, as well as people’s lack of awareness that AYB also produce tubers that can be equally used for consumption, further contributed in the decrease of interest in AYB and hence the erosion of its useful genetic resources compared with other legume crops [6]. Despite these facts, recent data suggests that AYB is a good source of several mineral nutrients such as iron (Fe), zinc (Zn), magnesium (Mg), potassium (K) and calcium (Ca) [7, 8]. It is noteworthy to mention that AYB possesses higher amino acid content than those in other legume crops such as pigeon pea (*Cajanus cajan*), cowpea (*Vigna unguiculata*) or bambara groundnut (*Vigna subterranea*), and the levels of essential amino acids such as lysine, methionine, histidine, and iso-leucine contents are equivalent to levels found in soybean [5, 6, 9, 10]. Being a traditional crop with nitrogen-fixating capability, AYB has tremendous potential to address environmental concerns of sub-Saharan Africa (SSA) region such as soil degradation, where AYB could be utilized to improve soil fertility.

Currently, in-situ conservation of AYB seeds is practiced mainly by a few traditional farmers who value the crop’s cultivation [11] as well in germplasm banks, including the IITA Genetic Resources Centre (GRC) at Ibadan, Nigeria. For conservation purposes, the organized exploration and collection of AYB germplasm was believed to be distributed across north-east, east, west-central, west and south tropical Africa [12, 13], but currently, there is an urgent need for AYB information update, biodiversity conservation and maintenance.

**Fig. 1:**
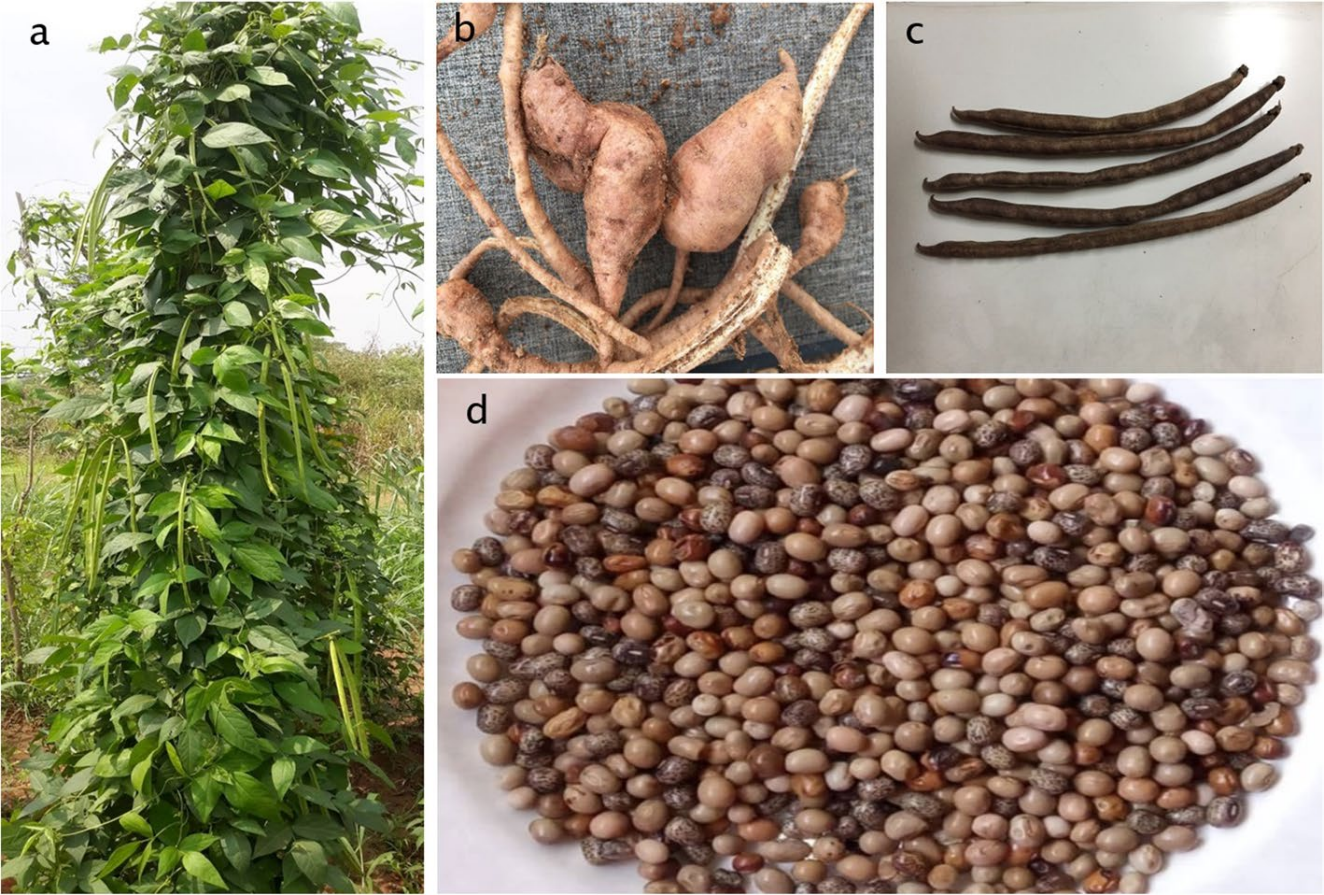
Morphological traits of AYB. **a** An adult plant of AYB with long green pods, **b** tubers, **c** dry pods, **d** seeds (*Photos from Ademola Aina*)

The diversity of AYB, in terms of morphological traits (Fig. 1), has been used in previous studies to group the various existing accessions into distinct groups based on phenotypic data [4, 14–18] as well as the description of significant genetic variability divided into different clusters and sub-clusters. Evaluation of the genotypic and phenotypic intraspecific variability in AYB accessions divided the genotypes into four to six clusters [1, 5, 6, 14, 15, 17, 19]. More recently, Aina et al. [18] described the phenotypic variability within 50 accessions of AYB collected within Nigeria and found that seed shape and cavity ridges on pods were the most discriminatory traits in terms of morphological characters. In his study, based on seed eye color pattern, AYB accessions were separated into two main groups, namely, accessions having white or grey testa with incision-like eye pattern and accessions having brown testa with varying degrees of incision eye pattern [18]. Even though morphological characterization is important and useful, it could be misleading as these traits are influenced by environmental conditions [20]. Consequently, several diversity studies have been done on selected AYB accessions using different molecular markers, including random amplified polymorphic DNA (RAPD) [21], amplified fragment length polymorphisms (AFLPs) [22, 23] and simple sequence repeat (SSR) markers [1]. Over the years, next-generation sequencing (NGS) technologies have emerged as quick and inexpensive approaches to study genetic diversity in crops [24, 25]. Among the several NGS approaches based on genotyping by sequencing (GBS) methods, some techniques rely on the genome complexity reduction using restriction enzymes [26]. For example, RAD-Seq approach relies on sequencing of small fractions of the genome and subsequently, genetic markers are identified across the genome. The cost effectiveness of the RAD-Seq over other NGS based methods cannot be overemphasized as the use of restriction enzymes coupled with barcoding increases the efficiency of multiplexing allowing the genotyping of multiple or different populations at the same time [27]. In terms of breeding purposes and targeting the African continent, high dry matter content and associated traits has been defined as key traits [28] which greatly depends on the genetic background of AYB [29]. Higher contents of dry matter in AYB tubers would increase competitivity and adoption against other root and tuber crops especially cassava and yam. Therefore, the objectives of this research were to assess the genetic diversity and to understand the genetic structure of 93 AYB accessions maintained at the IITA Genebank using RAD-Seq technology. Moreover, there was an attempt to correlate the phenotypic and genotypic variability of the studied accessions. To our best knowledge, this is the first report of SNP genotyping correlated with phenotypic descriptors in African yam bean for conservation and breeding proposes.

## Results

### Genetic diversity summary statistics

A summary of the diversity index statistics of the RAD-SNP markers is presented in Table 1. For the 3722 RAD SNP markers, the minor allele frequency (MAF) ranged from 0.017 to 0.500 with an average of 0.296, whereas the major allele frequency (MaF) averaged 0.704 and ranged between 0.500 to 0.983. The polymorphic information content (PIC) varied from 0.033 to 0.375 with an average of 0.288. The heterozygosity varied from 0.000 to 1.000 with a mean value of 0.416, whereas the gene diversity ranged from 0.034 to 0.500 with an average of 0.365.

**Table 1:** Diversity indices statistics of 93 accessions of AYB based on 3722 SNP markers

	MaF	MAF	GD	He	PIC
Maximum	0.983	0.500	0.500	1.000	0.375
Minimum	0.500	0.017	0.034	0.000	0.033
Mean	0.704	0.296	0.365	0.416	0.288

*MaF* Major allele frequency, *GD* gene diversity, *He* Heterozygosity, *PIC* polymorphic information content, *MAF* Minor allele frequency

### Genetic distance, population structure and cluster analysis

The genetic distance between accessions varied from 0.103 to 0.524 with an overall average distance of 0.369 (Additional file 2a). The majority of genetic distances (86.1%) was observed to be within the interval of 0.3001 and 0.4200 (Additional file 2b). The minimum genetic distance was observed between accessions TSs5A and TSs100 whereas the maximum value for this measure was found between TSs44 and TSs47 (Additional file 2a).

**Fig. 2:**
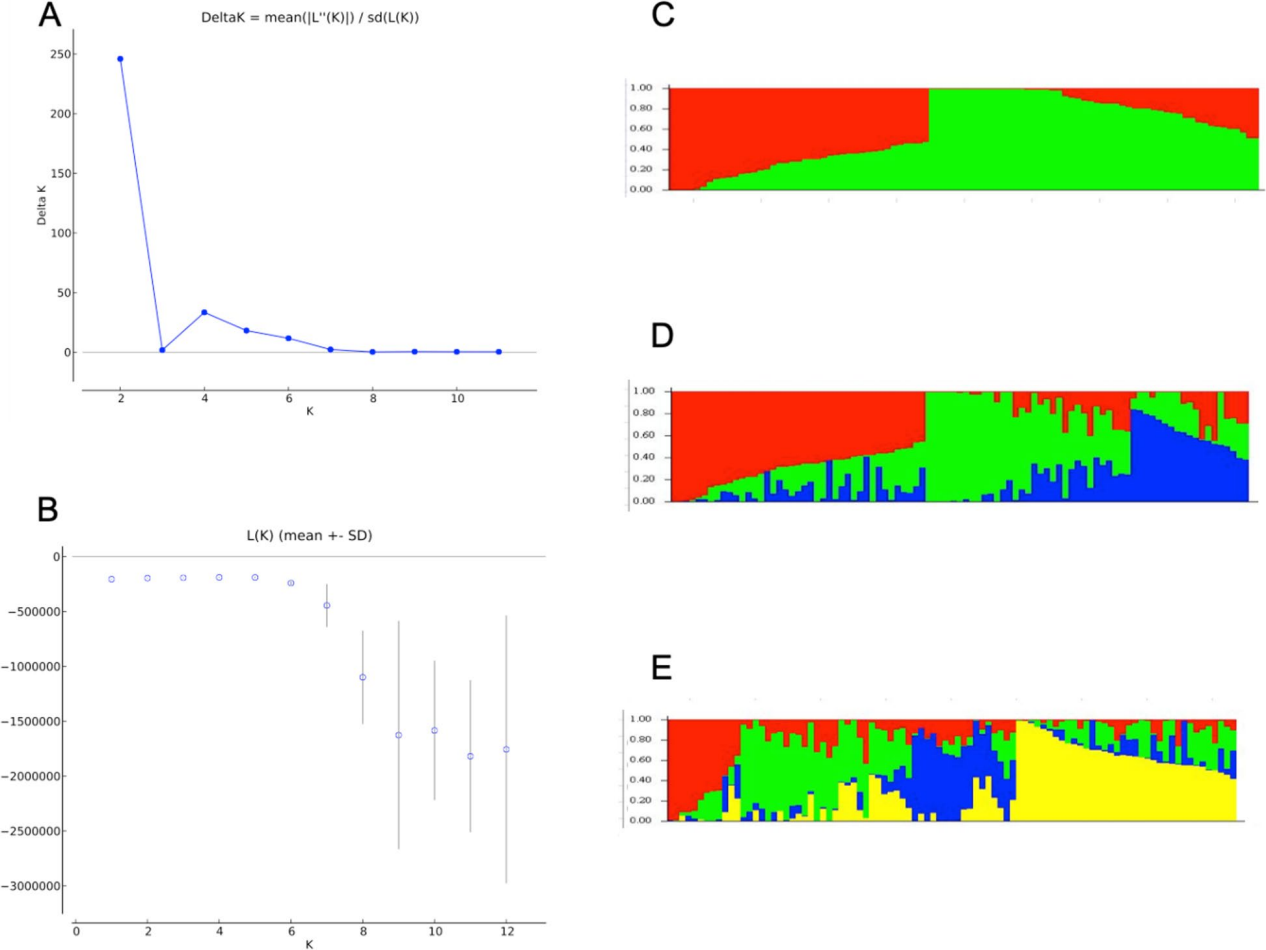
Graphical representations of the Bayesian Information Criterion (**A** & **B)** versus number of clusters and clustering algorithm, **C**, **D**, and **E** Admixture ancestry cluster at K = 2, K = 3 and K = 4, respectively

To visualize the pattern of variation among the AYB accessions, complimentary methodologies, namely Bayesian-based model implemented in STRUCTURE together with discriminant analysis of principal components (DAPC), PCA analysis, and PCoA analysis were employed. The ΔK statistic, calculated in STRUCTURE HARVESTER based on Bayesian clustering algorithm implemented in the STRUCTURE, indicated the best value of K to be 2 (K = 2) followed by a minor peak at 4 (K = 4) (Fig. 2) anticipating that the present set of AYB accessions would be divided into either 2 or 4 sub-populations. At K = 2 with membership probability of higher than 80%, a total of 11 and 30 accessions were assigned to Cluster 1 (red color) and 2 (green color), respectively, while remaining 52 lines were considered as admixed (Additional file 3). However, this clustering (K = 2) did not explain the phenotypic variability found in the AYB panel. Based on the small peak appeared at K = 4, the accessions were divided into four different clusters, suggesting 6, 7, 10 and 4 accessions in first (C1, red), second (C2, green), third (C3, blue) and fourth (C4, yellow) clusters, respectively. While remaining 65 accessions showing membership probability less than 80% were considered as admixed (Additional file 3). When considered value of K = 3, 14 accessions fell in first (C1, red), 15 accessions into the second (C2, green) and 4 accessions into the third (C3, blue) cluster. A fourth (C4, black color) cluster with mixed individuals was formed with 60 accessions.

.

**Fig. 3:**
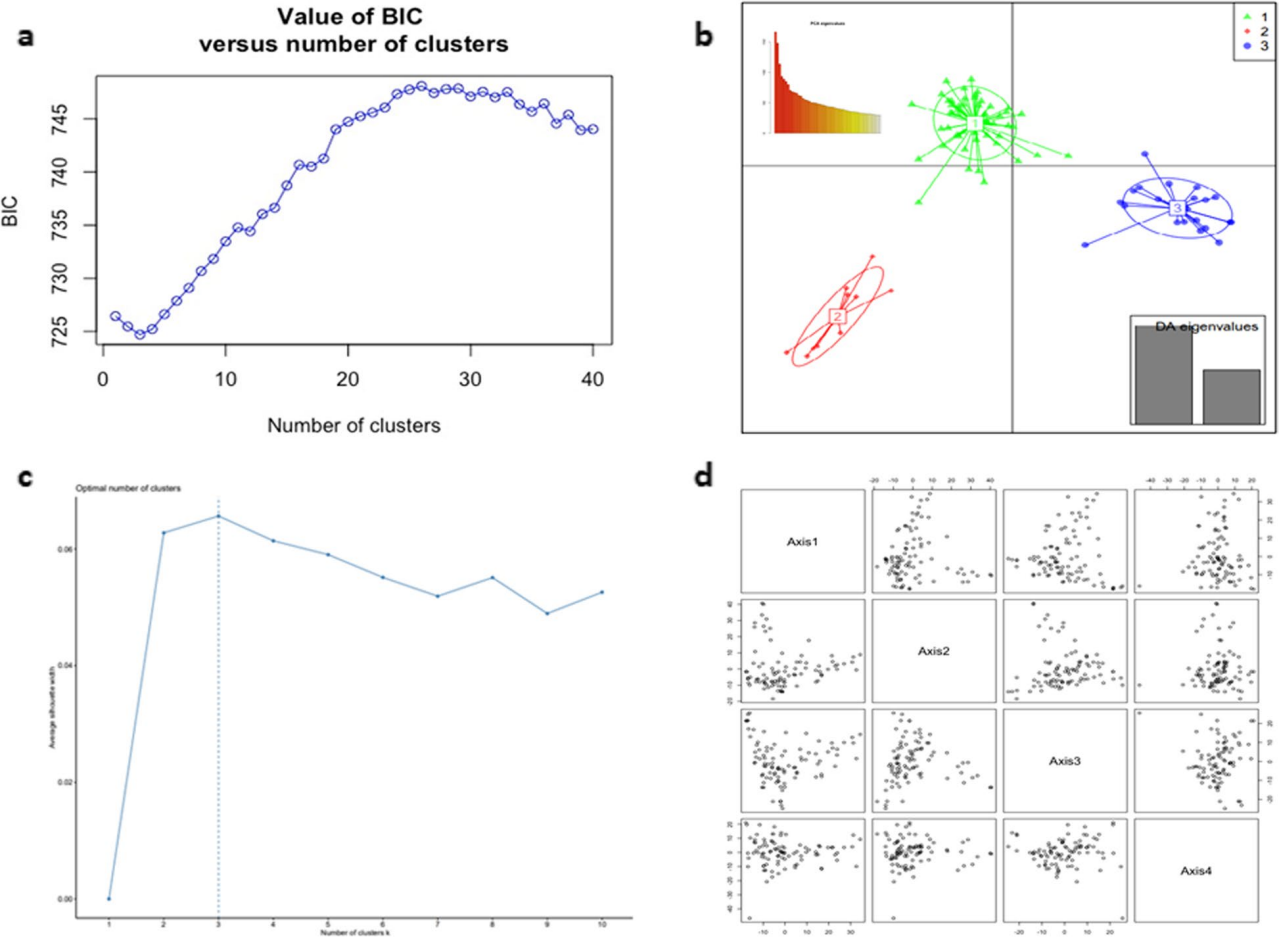
Graphical results of discriminant analysis of principal components (DAPC) and PCA analysis for the 93 AYB accessions. **a** Bayesian information criterion (BIC) value versus number of clusters indicating a rapid decline from K = 1 to K = 3 and point that K = 3 would be the best number of clusters for this set of individuals, **b** Silhoutte plot, **c** DAPC scatter plot with eigenvalues graph embedded within; and **d** PCA plink mean denoting clustering for axis 1 to axis 4 and showing the same three clusters for axis 1 and axis 2, axis 2 and axis 3 and axis 1 and axis 3

Together with DAPC and PCA analysis, K = 3 was depicted as the best number of sub-populations present in the set, as shown by both Bayesian information criterion (BIC) values (Fig. 3A) and optimal number of clusters obtained by the silhouete plot (Fig. 3B). The PCA results are in accordance that this set of individuals could be divided into 3 main clusters (Fig. 3C & D). According to the DAPC analysis, second cluster (C2) had the maximum number of genotypes recorded with 59 accessions, followed by first (C1, 24 accessions) and third (C3, 10 accessions) cluster. The percentage of variance and data distribution is shown by the eigenvalues graph (Fig. 3C).

### Phylogenetic relations and clustering

**Fig. 4:**
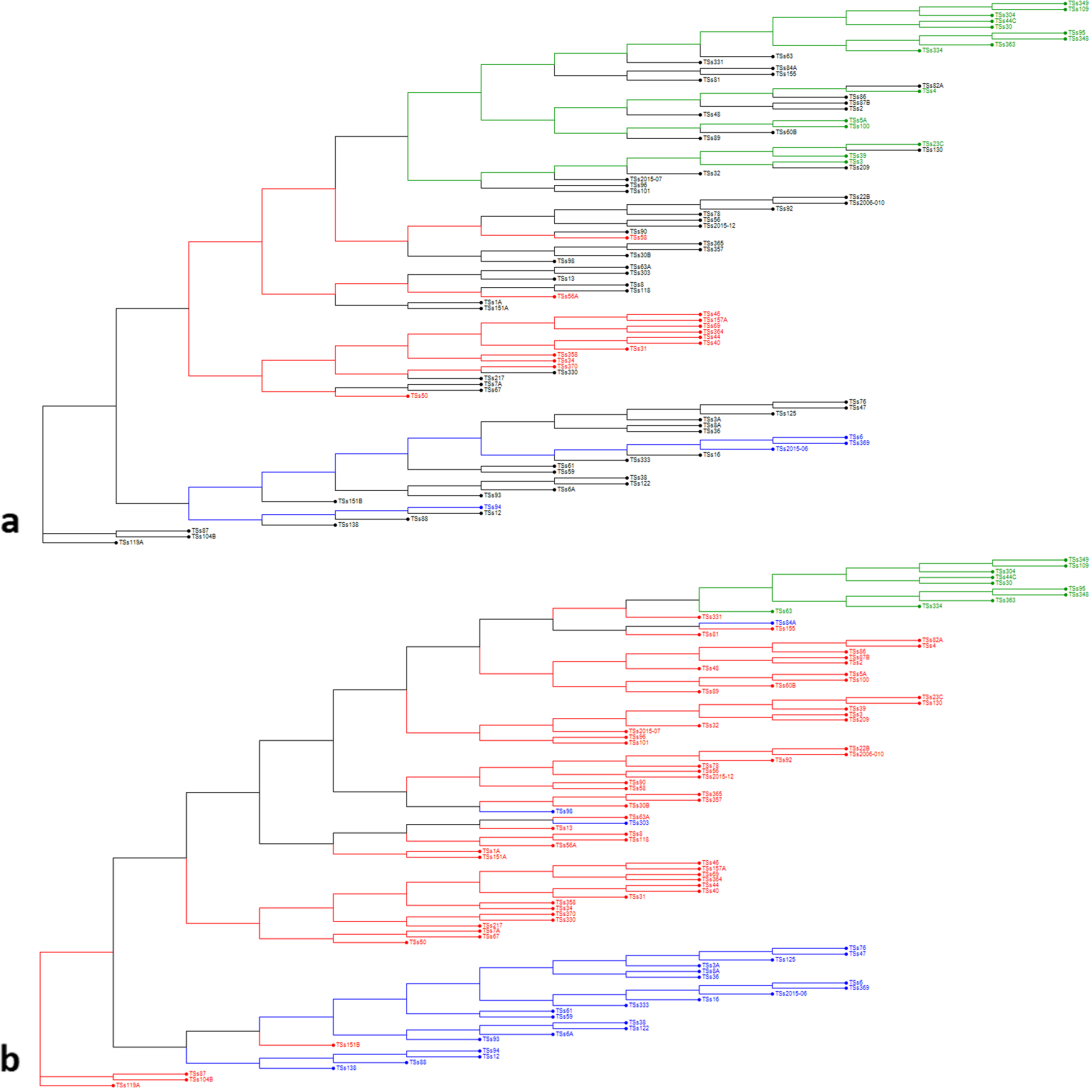
Phylogenetic relations of the sub-populations of 93 accessions of AYB based on SNP markers using **a**) the software DARwin at K = 3 clustering results from STRUCTURE and **b**) DAPC analysis. The red, green and blue colors represent the clusters 1, 2 and 3, respectively. The mixed individuals below the threshold level of 80% are represented with black color

Phylogenetic relations of the 93 AYB accessions indicated three main clusters (Fig. 4); a first cluster with only one AYB accession (TSs119A), a second cluster with two accessions (TSs87 and TSs104B), and a third cluster indicating presence of two sub-clusters where the majority of accessions (90) were included. Based on STRUCTURE results at K = 3, the three different clusters are well defined in the phylogenetic tree with the distinct STRUCTURE clusters being well identified in the different tree branches (Fig. 4a). Each accession was highlighted with blue (C1), red (C2) and green (C3) colors in the phylogenetic tree corresponding to their sub-populations suggested by STRUCTURE at K = 3. The accessions denoted with black color representing admixed genotypes, as the value of membership coefficient revealed by STRUCTURE was less than the threshold of 0.80. Nonetheless, the second-best possible K value suggested by STRUCTURE was 4 (K = 4), but this number of clusters do not depict the variability encountered in the population. Assigning the STRUCTURE results on the NJ tree at K = 3 (Fig. 4a), it is evident that the three different groups are separated distinctively (Fig. 2A).

Similarly, the DAPC analysis is also in good agreement with the clustering pattern of the neighbor-joining (NJ) and STRUCTURE (Additional file 4). All the accessions of first cluster (C1, 24 accessions) except TSs84A, TSs98 and TSs303 identified by DAPC corresponded with the NJ sub-cluster C3a while the accessions in sub-cluster 3b of NJ corresponded to the second and third cluster of DAPC (Additional file 5). Comparing the results of DAPC analysis with both NJ tree and STRUCTURE at K = 3, it gives a clear understanding of the grouping patterns of the 93 AYB accessions into three clusters (Fig. 4).

### PCoA analysis, FsT allele frequency, and variance among and within sub-populations

PCoA analysis performed by DARwin software [30] indicated that the first and second principal component axis described 8.3% and 5.6% of the variation, respectively. Together, both axes described 13.9% of the total variation (Additional File 6: Figure S2). Using STRUCTURE, the *FsT* found among clusters at K = 3 was of 0.223, 0.330 and 0.241 with expected heterozygosity of 0.347, 0.257 and 0.329 for clusters 1, 2 and 3, respectively. Analysis of molecular variance (AMOVA) among and within sub-populations, based on the 3722 SNP markers indicated a *FsT* value of 0.055 among the sub-populations. Among and within individuals, values of 0.069 and 0.010 were encountered for *Fis* and *Fit*, respectively (Table 2). For all the set of accessions a total value of 0.088 was found for *F’st*.

**Table 2:** Analysis of molecular variance among and within sub-populations^a^

Source	Df	SS	MS	% Est. Var.	F-Statistics	Value	P
Among Pops	2	5302.08	2651.041	5	Fst	0.055	0.001
Among Indiv	90	597,770.24	664.114	0	Fis	−0.069	0.001
Within Indiv	93	70,876.00	762.108	95	Fit	−0.010	0.001
Total	185	135,948.32		100	Fst	0.088	

^a^Based on 3722 SNP markers and 93 AYB accessions in DAPC analysis

### Phenotype-genotype clustering relation

**Fig. 5:**
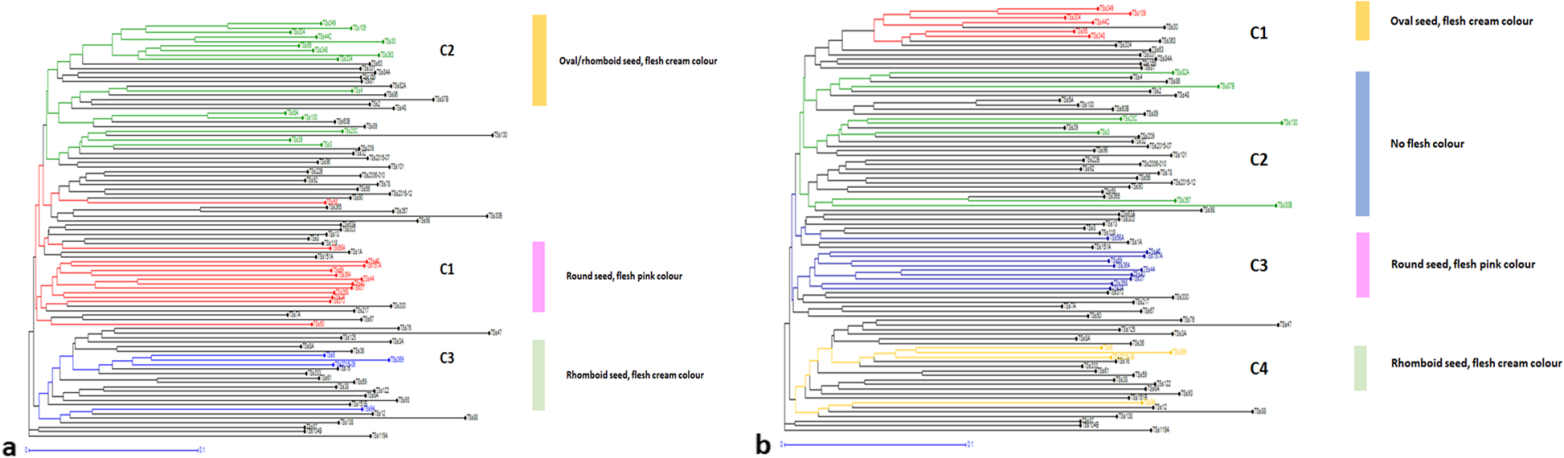
– Phenotype-genotype clustering relation considering K = 3 and K = 4 using STRUCTURE analysis at a 80% threshold. **a** At K = 3 the clusters 1, 2 and 3 are represented by red, green and blue colors, respectively. **b** At K = 4, clusters 1, 2, 3 and 4 are represented by red, green, blue and yellow colors, respectively. The mixture genotypes are represented by black color. To each cluster there is the correspondence of main phenotype trait and represented by same colors (yellow for genotypes with oval seed, and cream flesh color; blue for no flesh color; pink for round seed and pink flesh color; and green for rhomboid seed and cream flesh color) considering both K = 3 and k = 4

We wanted to further understand if there could be found a relationship between the best probable number of clusters and the phenotypic characteristics of the studied AYB accessions that fall within each cluster. We took into consideration the best number of clusters as K = 3 and K = 4. At K = 3, the AYB accessions could equally be clustered based on phenotypic traits, particularly seed shape and tuber skin color (Fig. 4; Additional file 1). With a probability threshold value ≥80% (K = 3), all the accessions in Cluster 1 had round seeds and tuber flesh of pink color except accessions TSs56A and TSs58 that were characterized by having brownish orange flesh color (Additional file 5). Among all the accession assigned in first cluster, only single accession TSs67, did not produced tubers but presented round seed shape. In the second cluster, majority of AYB accessions presented oval seed shape (71.5%), but rhomboid and round seed shapes were also noticed in four (TSs100, TSs39, TSs4 and TSs5A) and one (TSs44C) accessions, respectively. However, all accessions except TSs39 (non-tuber forming) produced tubers with flesh cream color. The third cluster comprised 4 individuals, all with rhomboid seed shape and no tuber formation. A great majority of accessions (60) were suggested as admixed. When considering DAPC analysis, all accessions were grouped into the 3 different clusters, but these clusters did not associate with phenotypic variability except second cluster (Additional file 4). If considered K = 4 as indicated by second best value of K detected by STRUCTURE, the grouping into four clusters is better adjusted to the phenotypic differences especially seed and tuber yield produced by accessions in present AYB panel. The first cluster contained accessions having both low seed and tuber yield except genotype TSs44C exhibiting medium seed yield (Fig. 5, Additional file 5). Interestingly, accessions in the second cluster exhibited high seed yield but no tuber formation with exception of TSs23C and TSs3 which also produced tubers with cream flesh color. Notably, all the accessions assigned in third cluster were observed better for both seed and tuber yield especially genotype TSs56A which produced high seed yield together with good tuber formation. In fourth cluster, none of the accessions produced tubers but showed wide range of seed yield. It is noteworthy to mention that either considering the three or four clusters (K = 3 or 4), accessions TSs59, TSs84A and TSs8A which produced maximum seed yield per plant (≥200 g) fell in admixed group (Additional file 6).

## Discussion

Assessment of genetic diversity based on either or both phenotypic and genotypic data is a crucial component for every crop improvement program, as new alleles may encode a variety of complex and superior traits ranging from resistance to biotic and abiotic stresses, superior organoleptic and nutritional characteristics. The utilization of genotyping greatly facilitates the successful grouping of germplasms into different clusters that assists to understand both existence of genetic variability and phylogenetic relationships among each of them. Results based on the dissimilarity further facilitates in choosing the most diverse genotypes for crossing resulting in higher probability to give superior hybrids [31].

In this study, the PIC values were much lower than previously reported studies in AYB [1, 19, 21–23] which are mainly attributed to the type of markers used because RAD-SNP markers are bi-allelic in nature when compared with multi-allelic markers [32]. Furthermore, such discrepancy in genetic diversity could also be due to the differences in the studied material and number of applied genotypes as well as markers [32–34]. However, the PIC values obtained in present study are similar to ones obtained from DArT SNP markers in maize [24] and sugar beet [35]. Similarly, the observed genetic distances were found within the range observed previously by other studies [21, 22], but very distinct than that of Shitta et al. [1] and Nnamani et al. [19], who described wide ranges based on the results of SSR and ISSR markers, respectively. The genetic distances recorded in this study, suggest the presence of substantial genetic diversity in AYB germplasm compared to other legumes like cowpea mini-core collection maintained at IITA [25]. This is also supported by the values of heterozygosity (He) obtained among the accessions (Table 1).

Multiple clustering methods (Bayesian vs. multivariate analysis) were used in the present study to truly understand the population genetic structure of 93 AYB accessions because single method may lead to biased assessment of data. The results obtained herein, were also implemented in the Neighbour-joining phylogenetic tree taking in account a cutting-off of 90%. Despite a highly stringent cutting-off value, it gives an assurance of the results obtained. The Bayesian clustering-algorithm STRUCTURE identified two groups (K = 2) as best results for clustering in the present set of AYB accessions, nonetheless, a minor peak at K = 4 indicated the presence of 4 groups. It is not uncommon that sample sets subgrouping of individuals identifies best K as 2 [36] but it was not able to explain the variability shown by the 93 AYB accessions studied here. The DAPC method is a multivariate method that uses sequential K-means to infer the best number of genetic clusters. In this study, both DAPC and PCA identified the best K to be 3 (Fig. 3). All together based on genotypic data these methods indicated that the 93 AYB accessions can be clustered into three main clusters, as three genetic groups were continuously depicted by the different methods and level of clustering. These results do indicate a strong evidence of the genetic differentiation in the different groups of the AYB accessions. The major difference among clustering methodologies was the number of mixed lines found in the Bayesian admixture method, as in the DAPC method these lines were distributed among the different clusters (Additional file 5).

Regarding population differentiation, the fixation index (*F*_*ST*_) values estimated among populations (0.055), indicates a low degree of genetic differentiation between the populations. The negative values obtained for the non-random mating coefficient (*F*_*IS*_*)* among individuals, as well for the global mean inbreeding coefficient (F_IT_) within individuals, indicates that the genotypes are of outbred nature but with a high degree of inbreeding (Table 2). The positive but low value (0.088) of non-random inbreeding between subpopulations (F’_ST_) suggested substantial differentiation among the found populations and reveals a good source of materials for crop improvement.

In this work, we attempted to correlate the grouping of the accessions based on SNP markers and their corresponding phenotypes in AYB for the first time. The phenotypic traits were successfully associated with genetic clusters where great majority of accessions shared a similar phenotype, such as seed shape, tuber flesh color, seed, and tuber yield (Fig. 4, Additional file 1). The Bayesian STRUCTURE method was found more stringent than DAPC and suggested 64.5% of the genotypes as admixed (K = 3), nonetheless, DAPC method also allocated all the accessions into three clusters but without any admixture. Comparative analysis of STRUCTURE at K = 3 and K = 4, revealed consistent results with first and third clusters (Fig. 5; Additional files 5 and 6) but second cluster (at K = 3) was further partitioned into two different clusters (C1 and C2) when K value was considered as 4. Interestingly one of the groups (C1 at K = 4) is characterized as poor performer for both seed and tuber yield having oval seeds shape and cream flesh color except accession TSs44C showing round seed shape. Notably, the other group (C2 at K = 4) contained non-tuber forming accessions exhibiting high seed yield with various seed shape such oval, oblong or rhomboid seed shape except TSs23C and TSs3 which also produced very low amount of cream flesh color tubers. The accessions in third cluster (C3 at K = 4) corresponding to C1 cluster at K = 3, exhibited very high seed as well as tuber yield with round seed shape and pink flesh color except TSs56A which had brownish orange flesh color. The grouping of accession TSs69 in C3 cluster is in concordance with the results of Aremu et al. [37] who previously advocated the use of TSs69 for dual-purpose production capacity. It is noteworthy to mention that fourth cluster (C4 at K = 4) corresponding to C3 cluster at K = 3 contained non-tuber forming accessions with rhomboid seed shape but produced medium seed yield. Similarly with previous research works [1, 19, 22, 23], our results also indicate that the best K to be considered either K = 3 or K = 4, depending on phenotypic traits chosen.

In Africa particularly in Nigeria, depending on the region, AYB can be grown either for tuber or seed production, with the yield for tubers described as high as 0.5 kg per plant and average of seed yield per plant between 100 and 200 g [38]. The accessions in fourth cluster such as TSs2015–06 (high seed yield but no tuber formation) could be used in breeding for better seed yield purpose. In the past, it has been reported that both seed and tuber yield are inversely correlated [39]. It is noteworthy to mention that accessions such as TSs56A, TSs358 and TSs364 (both high seed and tuber yield) belonging to third cluster could be used in breeding program for dual purposes. Recently, accessions TSs56A and TSs34 identified suggest their potential utilization in AYB breeding program due to better seed yield and its component traits. Of the 93 AYB accessions, 69% genotypes (64 accessions) including the three best accessions (TSs84A, TSs59 and TSs8A) exhibiting seed yield greater than 200 g plant^− 1^ did not produce tubers (Additional file 1).

In yam bean (*Pachyrizhus tuberosus* L.), flower, stem and fruit pruning are a general practice to increase yield-related traits. For instance, Zanklan [40] observed that reproductive pruning of yam bean led to a great increase in fresh tuber yield including dry matter yield and explained by avoiding the nutrient and water competition between tuber and pod formation, yet a large variation was found among accessions. Similarly, the positive impact of reproductive pruning on fresh storage root yields has been also reported in American yam bean [41, 42]. Despite reproductive pruning is a common practice in the yam bean including Mexican and Andean yam bean, more studies are needed in AYB because there are evidences of either adverse or independent effect of pruning and genotype on tuber fresh weight but also interact for tuber dry matter [43, 44].

## Conclusions

For the first time, we assessed the genetic diversity of AYB using SNP markers and relate the genetic clustering of accessions with their phenotype. The best number of clusters were found to be either 3 or 4 depending on trait specific analysis. We could separate clusters based on agronomic traits, indicating that these traits can be used in future for breeding-decision making and conservation purposes. Based on the assessment of agronomic performance and their corresponding genotypic results, these AYB accessions could be utilized in breeding programs either for seed yield (TSs2015–06; cluster C4) or both seed and tuber yield (TSs56A, TSs358 and TSs364 for high seed and better tuber yield; TSs34 and TSs44 for high tuber and good seed yield; cluster C3) and/or subsistence farming (TSs23C medium seed as well as tuber yield; cluster C2). Additional field evaluation with global positioning system (GPS) to trace the exact origin of collection together with full-genome sequencing and annotation of AYB will be of great use to further tap the diversity in AYB.

## Materials and methods

### Germplasm description, plant growth and phenotypic data

A total of 93 AYB accessions were collected from the Genetic Resources Center (GRC), International Institute of Tropical Agriculture (IITA), Ibadan, Nigeria. The accessions used in present AYB panel originated from diverse sources which were collected and conserved in IITA gene bank along the last 20 years (Additional file 1). For genotyping, the plant materials were transported under the guidelines of the United States Department of Agriculture, Animal and Plant Health Inspection Service, USA, and grown under screen house conditions at the University of California, Davis, USA. For phenotyping, the accessions were evaluated at IITA resource nursery, Ibadan, Nigeria [7^o^ 23′ 16“ North (7.39^o^) 3^o^ 51’ 30” East (3.86^o^)] during the season of 2020.

### DNA extraction and rad-Seq sequencing

Leaf samples of two-weeks old plants were collected for DNA extraction and kept at − 80 °C until use. Prior to genomic DNA extraction, samples were lyophilized (Labconco, USA) and subsequently ground in a spex™Sample Prep 2000 Geno/Grinder (Thomas Scientific, USA). Genomic DNA was extracted from the ground leaves of each accession using commercial QIAGEN DNeasy Plant Mini Kits according to the manufacturer’s protocol (Qiagen, Germany). The DNA purity and quantity in each sample were checked on the NanoDrop spectrophotometer (ND-1000) (Thermo Fisher Scientific, USA) and subsequently, confirmed on a 1% agarose gel stained with SYBR green (Thermo Fisher Scientific, USA). African yam bean DNA library was constructed using the double digest RAD protocol [45] which relies on the use of restriction enzymes to reduce genome complexity and to improve the efficiency and accuracy of SNP genotyping. The quantification (quality and quantity) of the samples was performed using a bioanalyzer DNA Sensitivity chip (Agilent Technologies, Santa Clara, CA). Thereafter, single end sequencing was performed on a Hiseq 3000 platform (Illumina, San Diego, CA) at the UC Davis Genome Center, California, USA.

### Data cleaning and analysis

A total of 393,414,882 reads was generated from the sequencing of the 93 AYB accessions panel using the Illumina RADseq RAD markers. Of the 43,061 RAD tags identified by the Tassel’s UNEAK Pipeline, only the RAD tag pairs differentiated by one SNP were depicted. The raw file was converted into Powermarker V3.25 [46] format for statistics summary calculation. All markers that had a MAF value ≤0.01 and missing data ≥10% were eliminated [24, 47]. Finally, 3722 RAD markers were selected and used for further analysis. The raw data has been deposited into NCBI and can be accessed through the following link https://www.ncbi.nlm.nih.gov/bioproject/PRJNA389330.

### Genetic diversity analysis and PcoA

The genetic diversity analysis of the 93 AYB accessions was performed using 3722 SNP RAD markers. The key measures of genetic diversity, including the genetic diversity (GD), polymorphism information content (PIC), heterozygocity (He) and minor allele frequency (MAF) were computed using the PowerMarker version 3.2.5 software [46]. The principal coordinate analysis (PCoA) was performed from the genetic distance matrix based on dissimilarity analysis results (Supplementary file 3) obtained from DARwin v.6.0.013 software (http://darwin.cirad.fr) [30] using 30,000 bootstraps.

### Structure analysis, principal component analysis (PCA) and allele frequency among clusters

Different complementary methods, ADMIXTURE model incorporated in STRUCTURE [48], discriminant analysis of principal components (DAPC) [49, 50], and principal component analysis (PCA) incorporated in the R software [51] were employed to study the pattern of population structure. To assign all the 93 AYB accessions into specific groups or clusters (without prior information of population) all the 3722 RAD SNP markers were firstly imported into the Bayesian Markov chain Monte Carlo software STRUCTURE v2.3.4. following inference of best lambda (λ) value using STRUCTURE software, the λ value was set as 1. For the choice of most probable number of K’s, simulations were initially performed by running a k = 20 (from 2 to 20) with 20 interactions, thereafter, the final analysis was performed using the admixture model, correlated allele frequency for each of the K’s using 10 runs in a 10,000 burn-in and 500,000 repetitions. The results obtained from STRUCTURE were imputed into STRUCTURE HARVESTER [52] to determine and visualize the best K. Based on the best LnP(P) of STRUCTURE, the ad hoc statistic ΔK was used in the determination of the optimal number of sub-groups or clusters [53]. The accessions presenting a membership coefficient value higher than 0.80 were assigned to a specific cluster or group. While remaining individuals with membership coefficient values lower than this threshold (< 0.80) were considered as admixted and assigned to an additional cluster. The allele frequency among the different clusters was obtained by STRUCTURE software.

To cross-check the results obtained by STRUCTURE, DAPC analysis was performed using the *find.clusters* function of the adegenet package [49] in R statistical software [51]. The total variance of a variable was partitioned into between– and within–groups and the value with least corresponding Bayesian information Criterion (BIC) was chosen as the best number of sub-populations. To further support our results, principal component analysis (PCA) was additionally calculated. The genetic distance matrix among accessions was obtained using DARwin software as described previously [30].

### Phylogeny studies

Population relationships were studied by imputing the full clean set of data into DARwin software [30] using neighbor-joining (NJ) tree feature by running 30,000 bootstraps. Results obtained from STRUCTURE and DAPC analysis were highlighted in the phylogenetic tree using specific colors in the phylogenetic tree, in correspondence to the different clusters using a final threshold value of membership coefficient higher than 0.80.

### Statistics

Analysis of molecular variance (AMOVA) was performed on DAPC population groups using GenAlEX software [54].

## Supplementary Information

**Additional file 1.** List of the 93 used AYB lines and phenotypic related traits.

**Additional file 2.** Genetic distance matrix.

**Additional file 3.** Structure results at K = 2, K = 3 and K = 4.

**Additional file 4.** DAPC analysis results.

**Additional file 5.** STRUCTURE vs DAPC genotypic-phenotypic traits.

**Additional file 6: Figure S1**. PCoA results; **Figure S2**. PCoA at K = 3 using 80% threshold.

**Additional file 7.** STRUCTURE results at K = 4. (PPTX 330 kb)

## Data Availability

The plant materials were grown at the IITA resource nursery which are available upon request to the IITA Genetic Resources Center (GRC). The raw sequence data herein reported have been deposited at the NCBI and can be accessed through the following link https://www.ncbi.nlm.nih.gov/bioproject/PRJNA389330.

## Abbreviations

BIC: Bayesian Information Criterion
AYB: African Yam Bean
DAPC: Discriminant Analysis of Principal Components
FiS, FiT, FsT: Fixation Indexes
GBS: Genotyping-by-Sequencing
GD: Genetic Diversity
GRC: Genetic Resources Center
IITA: International Institute of Tropical Agriculture
MAF: Major Allele Frequency
MaF: Minor Alelle Frequency
NGS: Next-Generation Sequencing
PCA: Principal Component Analysis
PIC: Polymorphism Information Content
RAD-Seq: Restriction-Site Associated DNA Sequencing
SNP: Single Nucleotide Polymorphism
