# Supplementary figures and images for: Predictive genotype-phenotype relations using genetic diversity in African yam bean (Sphenostylis stenocarpa (Hochst. ex. A. Rich) Harms)
Source: BMC Plant Biol. 2021 Nov 20;21:547. doi: 10.1186/s12870-021-03302-0 (PMC8605586; doi:10.1186/s12870-021-03302-0)

## Slide 1
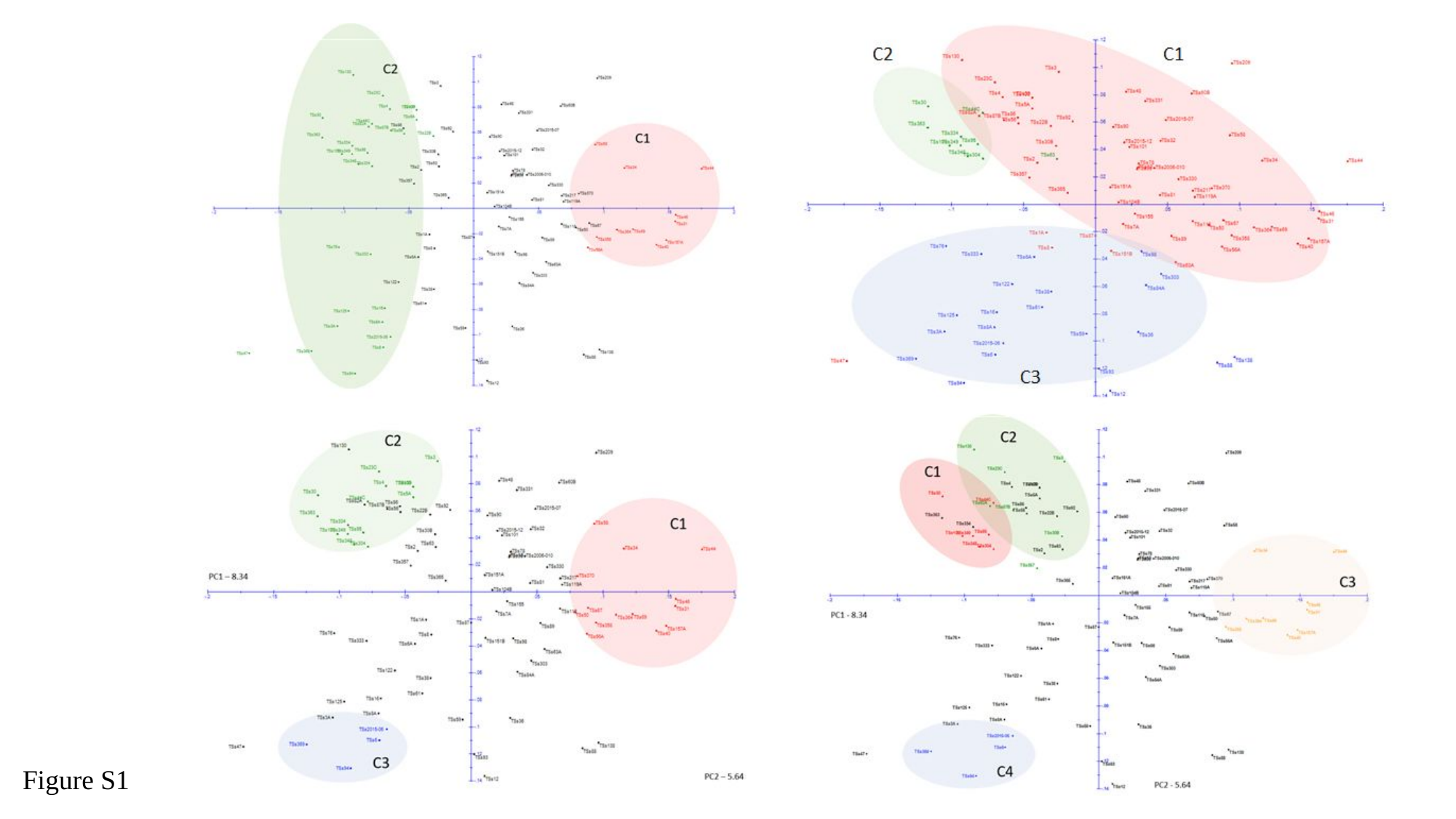

Figure S1

## Slide 2
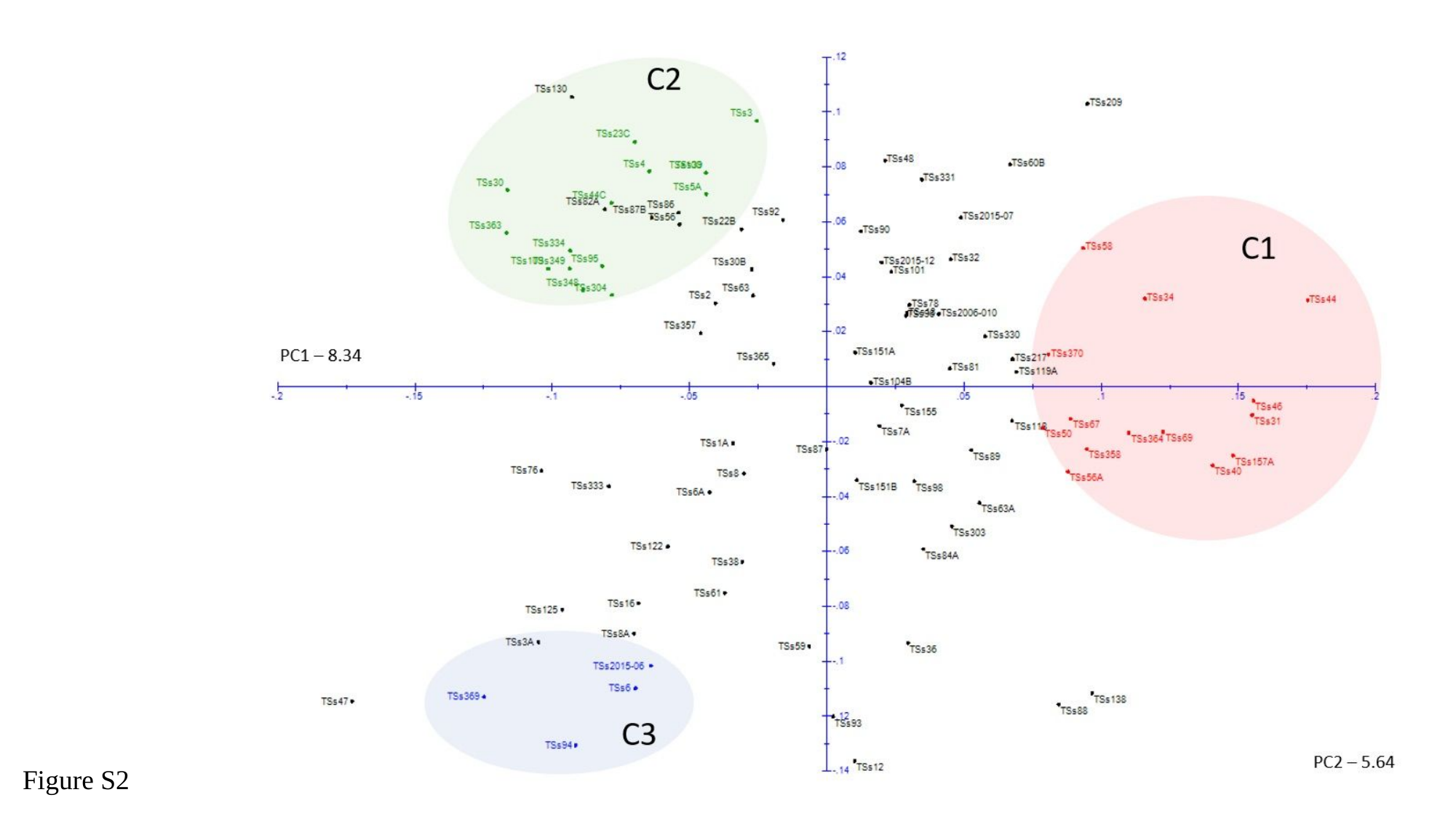

Figure S2

Supplement: Supplementary file 7 — Additional file 7. STRUCTURE results at K = 4. (PPTX 330 kb) [file 12870_2021_3302_MOESM7_ESM.pptx]
